# An analysis of design strategies for circular economy through life cycle assessment

**DOI:** 10.1007/s10661-022-09803-1

**Published:** 2022-02-14

**Authors:** Christian Spreafico

**Affiliations:** Department of Management, Information and Production Engineering, University of Bergamo, Via Marconi 5, 24044 Dalmine, Bergamo, Italy

**Keywords:** Design strategies, Circular economy, Life cycle assessment (LCA), Eco-design, Literature review

## Abstract

The goal of pursuing the circular economy (CE) is spreading more and more in industry, also driven by the introduction of new regulations, considerably affecting product design. However, a quantitative and rigorous evaluation of the environmental impacts of the results obtained by different design strategies used to implementing CE is missing in the literature. Those available only evaluate certain aspects of the life cycle of few products, belonging to specific application fields, in a qualitative way or they refer only to the global warming potential. This study provides a quantitative assessment of the environmental impacts reductions arising from the application of some common design strategies for implementing different CE options (e.g. reuse, waste to energy, remanufacturing), by using some standard indicators. The results were obtained by manually analysing 156 selected case studies of comparative life cycle assessment (LCA), extracted from 136 scientific articles. In them, the environmental impacts of design solutions for CE are compared with those of other solutions were wastes are not exploited. The obtained results have been used to evaluate the different design strategies for CE and to hierarchize them based on environmental sustainability of the solutions associated with them. In addition, an economic evaluation of the strategies, based on the life cycle costing methodology and exploiting the data available in the same articles, was also provided. Among the main achievements, it was found that the hierarchy of the CE options, pursued by the design strategies, to improve environmental sustainability is different from that provided by other studies. In addition, the environmental benefits associated with the different CE options strictly depend by the applied design strategies and the considered products.

**Graphical abstract:**

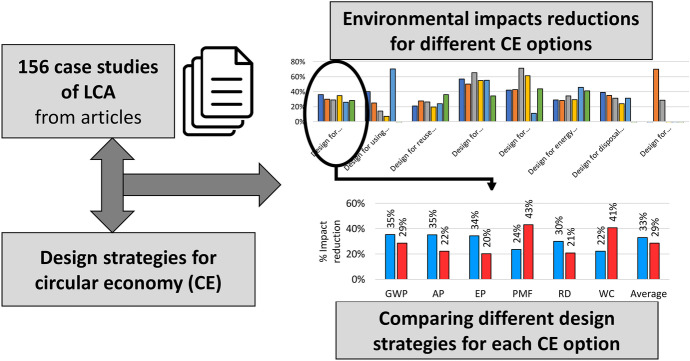

## Introduction

The implementation of the circular economy (CE) is increasingly becoming a fundamental requirement to be achieved during product design. As a result, the evaluation of the design solutions is significantly changing too in this direction and new aspects are considered. Products should be more robust to increase the operative life and reduce maintenance interventions (e.g. Van den Berg & Bakker, 2015). The same components must be more durable to be reused in new product after the disposal (e.g. Marino & Marrone, 2020). Any intervention on the product, during maintenance and disassembly, must be carried out by reducing the required energy and auxiliary materials. For this reason, the products should also be designed for the disposals as well as to guarantee the functioning (e.g. Rios et al., 2015). During the design, every aspect of the life cycle of a product must be seen as an opportunity, looking for its value. Consequently, an unusable product must be recycled as much as possible and energy must be obtained from the disposal of non-recyclable parts, by creating new synergies with the supply chain of the same and other products (e.g. Habagil et al., 2020; Yuan et al., 2014).

In the literature, some authors identified and classified strategies, methods and tools supporting product design to favour the transition to CE. These works highlighted the numerosity and the heterogeneity of the many supporting approaches and the criteria to be consider for selecting the most suitable ones (e.g. Bocken et al., 2016; Mestre & Cooper, 2017). Among them, there are the type of waste to be recovered, the type of product that generates or becomes the waste and the CE option to implement (e.g. reuse, recycle, remanufacturing). The pros and cons of different design approaches, emerged from these studies, also highlighted that an eco-design method cannot be considered better than a generic design method in absolute terms, and vice versa, in proposing solutions for CE—this despite the fact that the implementation of the CE requires to solve many of the problems that eco-design methods typically face (Den Hollander et al., 2017).

Thus, these studies, through environmental assessment, showed that merit of the higher sustainability of CE options depends by the application of the supporting design strategies. In fact, some preferential hierarchies between the different CE options regarding sustainability were provided (e.g. Behrens et al., 2007), but the application of different design strategies could make a CE option more sustainable than others, by reversing the hierarchy (Bocken et al., 2016). For this reason, the evaluation of the environmental benefits that the application of the different design strategies for CE can guarantee is therefore fundamental for eco-design and for implementing the same CE.

However, the studies supporting this assessment (see “State of the art”) have some limitations. The number of design strategies compared in each study is generally reduced. The considered scenarios and boundary conditions are too different, by making it difficult the normalization of the results. Because of the reduced number of considered case studies and their homogeneity, the design strategies are difficult to be evaluated in a domain-independent manner. The analyses have not always been carried out by rigorously following the reference standards. In many cases, the provided assessment is subjective and, consequently, they are difficult to compare. Finally, the impacts are usually expressed only through the carbon footprint.

This study proposes an evaluation of the environmental benefits arising from different products as a result of the application of different design strategies to support CE, which have been carefully selected in the literature. This assessment was conducted on 156 case studies from scientific articles based on life cycle assessment (LCA) according to ISO 14040 (ISO, 2006a) and ISO 14044 (ISO, 2006b). LCA methodology was considered due to its reliability in quantitatively evaluating the sustainability of the current technologies, critically discussing the choices to implement during eco-design and evaluating the environmental performances of new developed technologies (Hauschild et al., 2018).

The research questions to which this study aims to answer are the following:

- **RQ. 1:** *Is there a preferred hierarchy among the different CE options in terms of environmental sustainability, or does the hierarchy depend on the application of the different design strategies?*
- **RQ. 2:** *Can indications about how to apply the design strategies to obtain the greatest environmental benefits according to the addressed problem be isolated?*

The novelty and the importance of this studies lie in the method used to answer these questions. Compared to other studies in the literature, the evaluation of the design strategies, provided in this study, is less influenced by the specific features of the application fields. This is because more case studies, referring to a greater number of application fields, and with greater heterogeneity, were analysed. In addition, to reduce the influence of the design approach, followed to implement a CE option, alternative design strategies to achieve some of them were compared. The provided environmental assessment was improved, compared to the previous contributions from the literature, by expressing the environmental impacts through different standard categories and considering the entire life cycle of the analysed products.

These aspects allow to provide reliable and rigorous answers to the research questions and to confirm some considerations previously formulated by other studies in the literature. One of them is the official “waste hierarchy”, supported by studies (e.g. Zunft & Fröhlig, 2009) and reference bodies (e.g. European Union Council Directive 2008/09/EC). According to it, the CE options “reduce waste”, “reuse”, “remanufacturing”, “recycle” and “waste to energy” can be ordered on the basis of the environmental sustainability regardless of the application field. On the contrary, the obtained results could confirm if the same hierarchy is instead dependent on this latter and on the followed design strategies (e.g. Behrens et al., 2007). In addition, extended quantitative results about design strategies for CE can provide the knowledge base required by those studies that drawn the plan to teach CE (e.g. Saidani et al., 2021).

## State of the art

Several studies about the assessment of environmental sustainability achievable in a product through the application of design strategies for CE can be retrieved in the literature. By analysing those collected through the keywords “eco-assessment”, “product design”, “circular economy” and synonyms in Google Scholar and SCOPUS, the following differences emerge.

Some studies consider only the design strategies explicitly referred to CE. The most diffused ones suggest how to design a product to reduce waste, improve disassembly and recycle. Such strategies generally derive from generic design strategies (e.g. Cayzer et al., 2017) or from eco-design methods (e.g. Smol et al., 2017). Other studies (e.g. Boavida et al., 2020) analyse the effectiveness of design strategies, e.g. TRIZ, in the context of environmental sustainability but without referring to CE, although their insights can be used to implement it. Furthermore, depending on the case, the considered design strategies may refer to routine design (e.g. Garcia-Muiña et al., 2019), suggesting structural optimization and material replacement, or innovative design (e.g. Ozkeser, 2018), suggesting disruptive modalities to modify the product.

The design strategies were applied in different ways in these studies. In some cases, only a single one, while in other cases, at least a design strategy is considered for each CE option (e.g. Lieder et al., 2017). The main limitation of both these approaches is the comparison between the different design strategies. In contrast, the studies comparing multiple design strategies for each CE option do not have this limitation (e.g. Cayzer et al., 2017).

The number of case studies considered during the evaluations varies in the different contributions from the literature. Many papers provide a single case study about a specific application field (e.g. Moussa et al., 2019; Santagata et al., 2020); others consider more case studies but generally no more than ten, except for Spreafico (2021). The most difficult studies to compare are the ones they propose a single case study and a single design strategy (e.g. Liu et al., 2019).

The evaluation of the environmental benefits guaranteed on the product by the application of the design strategies can be carried out following a rigorous method, such as the LCA, by following its related standards (e.g. Garcia-Muiña et al., 2019), or other qualitative approaches of evaluation (e.g. Feniser et al., 2017).

Not always, the provided environmental impacts are expressed through standard categories. In the case, CO_2_ eq. (e.g. Smol et al., 2017) is the most common, although the use of this single indicator has been considered a limitation in quantifying the actual benefits of CE (Bocken et al., 2016). Non-standard categories are instead the reduction of the mass of the product and the produced waste (e.g. Boavida et al., 2020), the reduction of energy consumption (Ozkeser, 2018) and the reduction of the polluting emissions (Bersano et al., 2017).

Finally, regardless of the considered approach and environmental impact indicators, the provided assessment can be quantitative or qualitative. For instance, Cayzer et al. (2017) interviewed several managers of companies that had triggered a successful transition to CE by applying design strategies and provided their qualitative assessments.

## Tested design strategies for circular economy

With the aim of providing a broad overview of the many design strategies that can be used to implement the CE, in this study, a selection of those most discussed in the literature, for each CE option, was analysed. The considered CE options are reducing waste, using renewable energies, reuse, remanufacturing, recycling, product waste energy recovery, disposal, transforming waste into energy. In the following, a brief description of each strategy is proposed, along with the presentation of some sub-strategies with which these strategies can be declined. The starting point for retrieving the considered strategies was the work of Den Hollander et al. (2017). In that study, the authors collected generic design strategies and showed how their application can change in linear economy and in CE contexts, while the order of presentation of the design strategies is consistent with the hierarchy of EC directive 2008/09/EC (Zunft & Fröhlig, 2009) concerning the environmental sustainability of the different CE options (in descending order) to which the design strategies refer.

### Design for reducing wastes

Design for reducing wastes aims to ensure that the wastes generated from the product (i.e. exhaust components and auxiliary materials/consumables) during use and end of life are limited (Keys et al., 2000). In the literature, this strategy has been declined in different ways, which have also been considered in this study. Structural optimization (e.g. Russo & Rizzi, 2014) is used to reduce product mass through structural rearrangement, (acting on the shape) or microstructural rearrangement (acting on the internal organization) of the structure. Fluid dynamic optimization (Cheshmehzangi et al., 2017) is used to reduce the mass of a fluid by modifying its local or global fluid dynamic conditions (e.g., pressure, temperature, flow rate, turbulence, spatial distribution) while ensuring its functions (e.g., heat transfer, thrust). Finally, dematerialization is a more innovative way to solve the problem and it is also counted among the principles of TRIZ (Russian acronym for Theory of Inventive Problem Solving) (Altshuller, 1984), one of the best known methods to support technical problem-solving. Its objective is to eliminate the components of a product, delegating their functionality to other ones or to the external environment, or to replace them with a field. Two typical examples are self-cleaning glasses, exploiting catalytic elements activated by sunlight to avoid detergents and laser cutting to replace a sawcut.

### Design for using renewable energies

The goal of this strategy is to improve the product functioning by introducing dedicated technologies able to exploit renewable energy, by reducing the consumptions (e.g., Nakata et al., 2005). The prerequisites of this activity are the research of the renewable energy source and the definition of its compatibility with product functioning rhythms, e.g. the hourly profile in the case of solar energy.

### Design for reuse

Reuse is the simplest way to satisfy one of the three macro-objectives of the CE, i.e. keeping products and materials in use, by reusing the product and its materials, after the operative life, without transforming them. To put this strategy into practice, however, it may be necessary to intervene appropriately on the product, even redesigning its structure, through “Design for reuse” rules and suggestions. This activity within CE has the objective of encouraging the user to reuse the product for the same or other uses, by improving the resistance of the product’s structure (Hooton & Bickley, 2014). Alternatively, the structure can be improved to be more easily reconverted to possible future users’ needs (Torroja et al., 1997). From the environmental point of view, the product must also be designed to encourage the reuse on site in order to reduce the impacts of the relocation (Friedler, 2004).

### Design for remanufacturing

The goal of this strategy is to encourage the remanufacturing of a product after the operative life, so that it can be perform its functions again or perform other functions. According to this strategy, the remanufacturing operations are also improved by reducing energy and resource consumption to increase environmental sustainability (Nasr & Thurston, 2006). At the application level, the same strategy is implemented with the choice of the most appropriate technology to perform remanufacturing and the definition of the use modality (e.g. Haziri & Sundin, 2019).

### Design for recycling

The task of this strategy is to identify materials that can be recycled and use them to realize the product, without affecting its functionality, and facilitating at the same time product recycling, disassembly and other preliminary operations (Kriwet et al., 1995). At a general level, this strategy can be obtained in two different modalities, both evaluated in this study. They depend on the output of the recycling and the involved technologies. In the case of ecosystem restoration, the objective is to regenerate the biological cycles of various ecosystems (e.g. forests, fresh water, inland wetlands) through different processes (e.g. extraction of biochemical feedstock, farming and anaerobic digestion) (De Groot et al., 2013), while, in the case of technical recycling, the objective is to regenerate the technical cycles, by reproducing the product constituent materials (EC directive 2008/09/EC), such as in the case of recycled paper and plastic.

### Design for energy recovery

This strategy aims to improve the product by reducing the wasted energy through the introduction of devices dedicated to its recovery and reuse within or without the same product (Den Hollander et al., 2017). To implement the strategy, the modalities and the technologies to recover energy must be determined and implemented. Some examples are the heat exchangers for the hot fumes and the kinetic energy recovery system (KERS) for the recovery of kinetic energy in vehicles during braking. In this study, the environmental benefits of the Design for energy recovery were evaluated only through the thermal energy recovery.

### Design for disposal (using biodegradable materials)

The goal of this strategy is to reduce the environmental impacts of the product disassembly by improving the structure of the same product. For this reason, biodegradable materials decompose more quickly, emitting fewer pollutants and requiring fewer resources are preferred (Den Hollander et al., 2017). In general, the design directions starting from this strategy are the reduction of the quantity of material to be disposed and its replacement with a more easily disposable material. In this study, only the second option was considered to differentiate this strategy from “Design for reducing wastes” strategy. Biodegradable materials can be substituted to synthetic materials or used in combination with them (e.g., cement filled with rocks). They can be natural material or based on natural materials, such as bio-based polymers (Godavitarne et al., 2017).

### Design for recovering energy from waste

The objective of this strategy is to select the most appropriate processes and technologies to dispose the product, maximizing the obtained energy from its decomposition and reducing the generated pollutants. The product characteristics are carefully analysed to select the most appropriate disposal technology and the structure of the product can be modified to facilitate the previous disassembly (Den Hollander et al., 2017).

## Methodology

This section presents the methodology followed to evaluate the considered design strategies for CE. As in Spreafico (2021), the main assumption of the followed methodology is to base the analysis on case studies about comparative LCA collected from the literature instead of replicate LCA from scratch. In this case, two real options for treating waste were considered. The comparison between the second option and the first option was manually interpreted by the author as the application of a Design strategy for CE, among those presented in “Tested design strategies for circular economy”.

By considering LCA studies published in the literature, the comparison is enlarged, since many more case studies can be collected in a reasonable time. Another advantage of this choice is to improve the reliability of the results, since they are collected from peer-reviewed studies published in prestigious international journals. Finally, the choice to consider comparative LCA studies allowed to exclude the necessary subjective assumptions to compare the results of independent studies, based on different scenarios, boundaries conditions and functional units.

Each step of the followed methodology is explained in detail the following sub-sections.

### PHASE 1 — collecting the case studies from the literature

To ensure the reliability of the results, only articles published in indexed international peer-review journals about comparative LCA were considered in this study. These documents were searched in Scopus and Google Scholar databases, by using the following query (referred to Scopus syntax): “compar* AND (LCA OR (life W/ cycle W/ assessment))”. In addition, to provide an updated analysis, only the articles published since 2010 were considered. The intentional generality with which the query was formulated was necessary to retrieve all the relevant documents, given the vastness of the topic and the many ways to refer to the applications of design strategies for CE. To isolate the relevant documents, it was necessary to opt for a manual search, despite the burden required by the same. The manual search that made it possible to collect the relevant articles was conducted in title and abstract, searching for the actual proposal of comparative LCA and a summary mention of the application of one of the design strategies for CE described in “Tested design strategies for circular economy”.

Therefore, the previously selected documents were manually analysed throughout the text, applying a rigorous selection based on the following criteria.

1. At least two real options of waste processing are described and compared though LCA methodology.
2. The two options consider the same waste, which is processed, albeit in different ways, in the same operating scenario and their comparison is based on a unique functional unit.
3. The alternative waste management option is presented in an exhaustive manner, reporting all the features necessary to understand which design strategy for CE has been applied.
4. The baseline option actually represents a true scenario and not a pejorative comparison term that is used only to enhance in the comparison the characteristics of what is proposed in the articles, i.e. the second option. This control was performed by considering additional assessments about the baseline scenario of the given waste that were inferred from the scientific literature.
5. The environmental impacts of the two options are assessed using the same calculation procedure.
6. The results of the two LCAs of the two options are expressed according to the same impact categories.

The final pool, after the selection, counts 156 case studies, extracted from 136 articles.

### PHASE 2 — classifying the case studies according to the design strategies for circular economy

The classification of the case studies according to the considered design strategies for CE was manually performed. The reliability of this process and its repeatability are favoured by the precise definitions that have been collected for the design strategies and their many sub-strategies, reported in “Tested design strategies for circular economy”, and by the rigorous document selection process, presented in “PHASE 1 — Collecting the case studies from the literature”. Thanks to this process, all the considered case studies describe very clearly both the referred CE options (e.g. reuse, recycling) and the design strategies (or sub-strategies) that have been implemented.

### PHASE 3 — extracting data from the case studies

In this phase, the environmental impacts resulting from the life cycle of each considered option from the case studies were extracted. The selected impact categories are the most common in the literature and include both global and local effects affecting both environment and humans.

The selected impact categories are:

- Global warming potential (GWP), expressed in kg CO_2_ eq.
- Acidification potential (AP), expressed in kg SO_2_ eq.
- Eutrophication potential (EP), calculated as the arithmetic mean of terrestrial, fresh water and marine eutrophication and expressed in kg P eq.
- Particulate matter formation (PMF), considering the production of PM2.5 or PM10 or their arithmetic mean if both are available and expressed in grams per cubic metre.
- Resource depletion (RD), obtained from the arithmetic mean of mineral, fossil, and non-renewable resource depletion indicators, and expressed in MJ.
- Water consumption (WC), expressed in cubic metre.
- Average impact, calculated as the arithmetic mean of the percentage reduction (see PHASE 4) of all the considered indicators.

### PHASE 4 — quantifying the eco-sustainability of the design strategies for circular economy

The percentage reduction in each environmental impact category (*j*) associated with each design strategy for CE (*x*) was calculated as the arithmetic mean of the percentage reductions in the same impact category in all the (N) case studies referred to that Design strategy.

1 $$\mathrm{\%}\,{\text{I}_\text{j}\;\mathrm{reduction}}_{\,\mathrm{Strategy}\,\text{x}}= \frac{\sum_{\text{i}=1}^{\text{N}}{\mathrm{\%}\text{I}}_{\text{j,i}}\mathrm{reduction}}{\mathrm{N}}$$

In turn, the percentage reduction of the given environmental impact category (*j*) in a case study (*k*) was calculated as the difference of the environmental impact, of the same category, of option 2 and that of option 1, divided by the environmental impact of option 2. This latter is the one resulting from the application of a Design strategy for CE, according to the performed association, when compared to option 1.

2 $$\mathrm{\% }\,{\text{I}}_{\text{j,k}}\;\mathrm{ reduction}= \frac{{\text{I}}_{\text{j,k}}\left(\mathrm{option }2\right)-{\text{I}}_{\text{j,k}}\left(\mathrm{option }1\right)}{{\text{I}}_{\text{j,k}}\left(\mathrm{option }2\right)}$$

For instance, the paper of Agarski et al. (2017) compares the environmental impacts of catalyst fluid (option 1) and ultrasonic aerosol (option 2) to perform a chemical synthesis process. This case study was associated with the “Design for reducing waste” strategy because option 2 allows to eliminate the catalyst fluid that is instead used and disposed in option 1. While in the paper of Akhshik et al. (2017) the environmental impacts of two engine beauty covers made by different materials are compared: fiberglass (option 1) and bio-based material (option 2). This case study was associated with the “Design for disposal” strategy, because option 2 can be disposed more easily than option 1 and it is biodegradable. The complete list of the two options compared in each case study and the associated Design strategies for CE are reported in Table 4 in the Appendix.

The same method was also used to evaluate the economic convenience of the Design strategies for CE. In this case, the costs of the two options have been substituted to the environmental impacts in Eq. (2). These costs were also extracted from the considered articles and were obtained, in the same, through the life cycle assessment (LCC) methodology, which is the equivalent methodology of the LCA, used for evaluating the costs instead of the environmental impacts.

## Results and discussion

In this section, the obtained are presented and discussed in detail. A specific order was chosen for their presentation according to the increasing of the level of detail. In this way, the reader can initially learn the main results obtained by comparing all the tested design strategies for CE and then the motivations behind the results. For this reason, the presentation of the results goes into the merits of the individual design strategies and discusses the advantages and disadvantages of specific applications according to all the considered environmental impact categories.

Table 4 in the Appendix reports all the considered articles, the extracted case studies, their classification and the considered data about the percentage reductions of the impact categories and costs extracted from them.

**Fig. 1:**
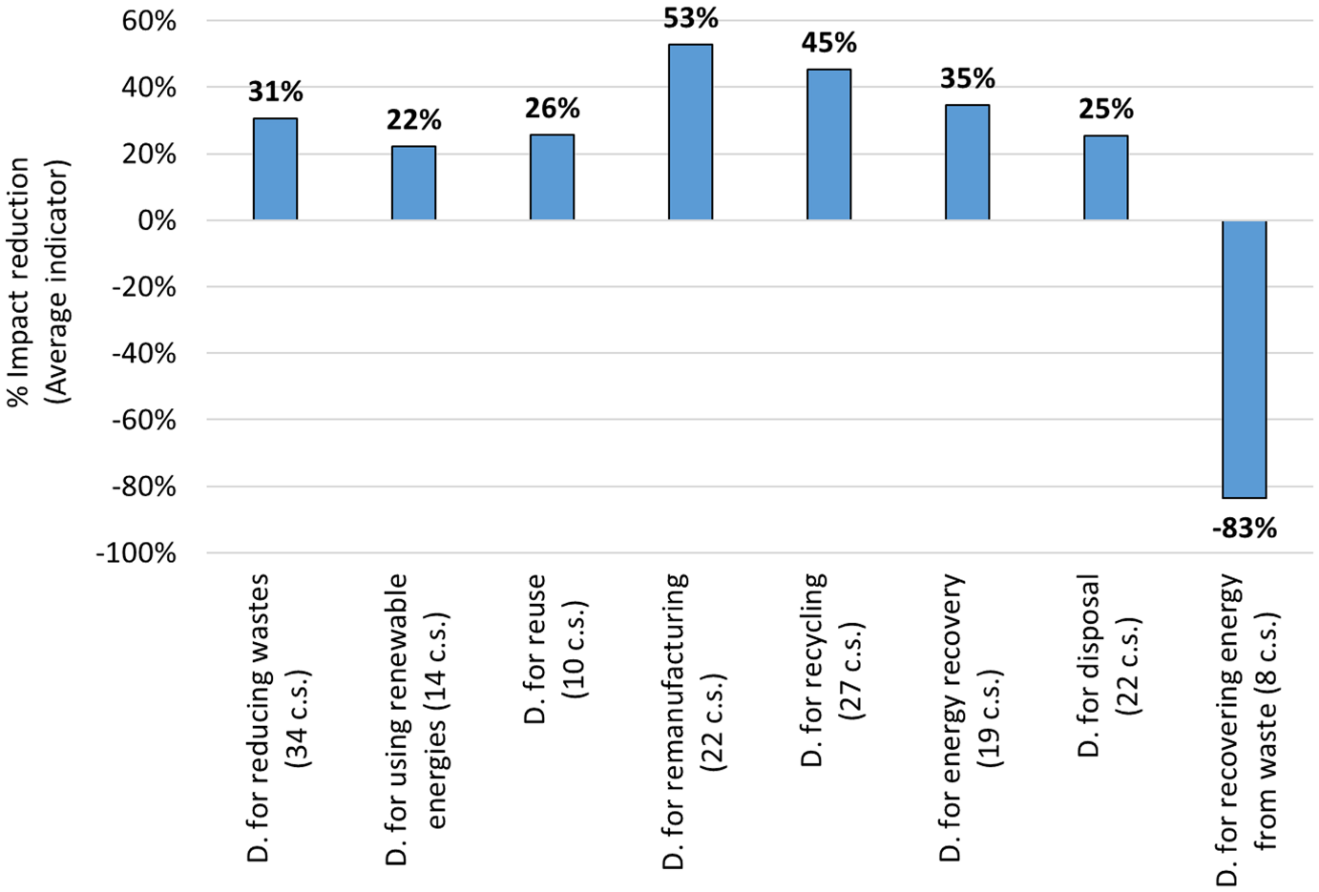
Percentage impact reductions of the average impact associated with each design (D.) strategy for CE resulted from the analysis of the considered case studies (c.s.)

The main result obtained from this study is the comparison of the different design strategies for CE, associated with the considered case studies, based on the average percentage reductions of environmental impacts (see Fig. 1).

**Fig. 2:**
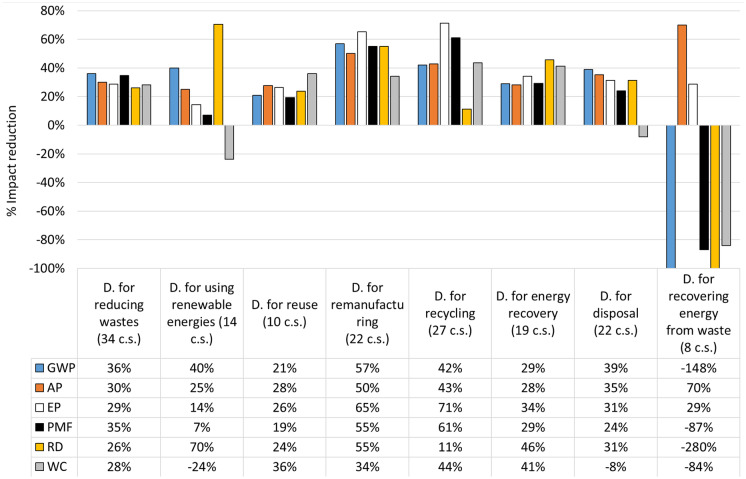
Percentage reductions of each environmental impact category associated with each design (D.) strategy for CE resulted from the considered case studies (c.s.)

Figure 1 clearly shows the environmental impact reduction of the Average indicator associated with Design for remanufacturing (53%) and Design for recycling (45%), and the disadvantages of Design for recovering energy from waste, where the average impact increased by 83%. However, this result only partially confirms RQ. 1 (i.e. “Is there a preferred hierarchy among the different CE options in terms of environmental sustainability, or does the hierarchy depend on the application of the different design strategies?”). In fact, Design for recovering energy from waste is the worst option, and a trend in reducing the average impact can be noted by moving from Design for manufacturing to this latter. However, the first three options do not follow this trend, contrarily to Zunft and Fröhlig (2009). Other studies (e.g. Amponsah et al., 2012; Cai & Waldmann, 2019) are aligned with this result: in them, reuse option is not more sustainable than recycling for certain materials. Anyway, the present study extended the perspective of this analysis that is generally limited to a certain application field in the other studies. To better understand the result of the average impact, Fig. 2 proposes the same comparison between the tested design strategies for CE, by providing all the considered environmental impact categories.

In addition, Table 1 provides the value of the standard deviations associated with the percentage reductions of the environmental impact categories for each considered design strategies (showed in Figs. 1 and 2).

**Table 1:** Standard deviations associated with the percentage reductions of the environmental impact categories for the considered design strategies for CE

	**GWP**	**AP**	**EP**	**PMF**	**RD**	**WC**	**Average impact**
**Design for reducing wastes**	30%	33%	33%	48%	23%	32%	33%
**Design for using renewable energies**	35%	46%	53%	55%	81%	23%	49%
**Design for reuse**	28%	33%	11%	4%	27%	12%	19%
**Design for remanufacturing**	27%	26%	32%	31%	37%	23%	29%
**Design for recycling**	94%	71%	63%	144%	78%	76%	88%
**Design for energy recovery**	28%	35%	30%	19%	39%	27%	30%
**Design for disposal**	32%	42%	23%	38%	43%	29%	34%
**Design for recovering energy from waste**	439%	131%	13%	90%	18%	460%	192%

By analysing Fig. 2, the categories of environmental impact that have most influenced the average results of each design strategy can be found. In the case of Design for remanufacturing, the reductions of all the categories are higher than the average, thus confirming the advantages of this strategy at a general level. The result of Design for recycling mainly benefited of the high reductions of EP, PMF and WC. Design for reducing wastes and Design for reuse have lower impact reduction values in all the impact categories. While Design for using renewable energies obtained the highest percentage reduction of RD and a good reduction of GWP, and a negative result of WC. Finally, Design for recovering energy from waste obtained the highest reduction of AP and negative results of RD, GWP and PMF.

In the following sections, the results obtained for each strategy are discussed in detail and according to each category of environmental impact, in order to identify the reasons about the method of application of the strategy and the application field.

### Design for reducing wastes

**Fig. 3:**
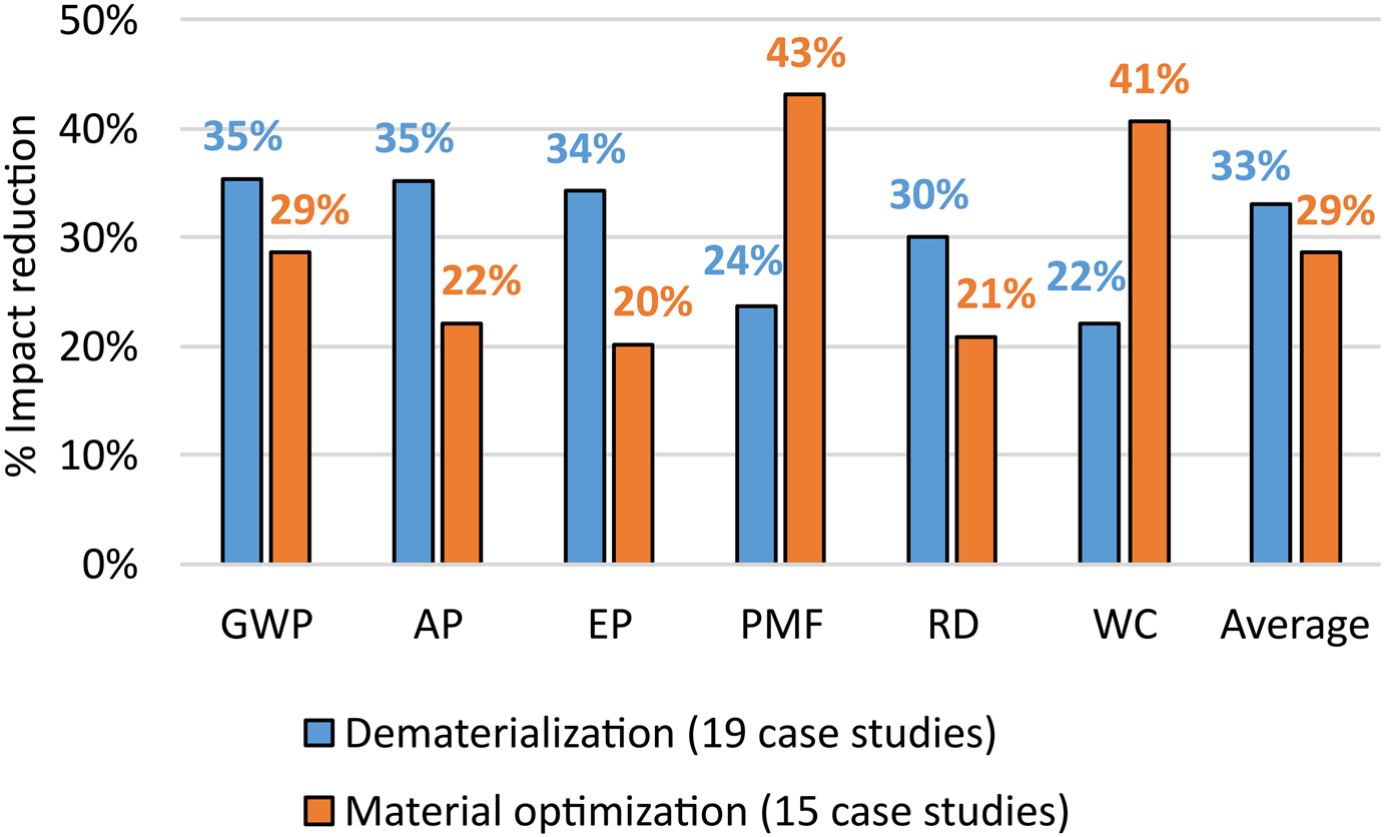
Percentage reductions of each environmental impact category associated with the two sub-strategies of Design for reducing waste

To understand the reasons of the results obtained for this design strategy, the case studies associated with it were divided between two sub-strategies: Material optimization (15 case studies) (e.g. Villanueva-Rey et al., 2018) and Dematerialization (19 case studies) (e.g. Joseph et al., 2015). Figure 3 shows the percentage reductions of the environmental impact categories that have been associated with these sub-strategies in the considered case studies.

Analysing Fig. 3, Dematerialization resulted batter than Material optimization, by allowing a higher percentage reduction (4%) of the average impact. This result was favoured in particular by the higher reduction of GWP, AP, EP and RD of Dematerialization, while Material optimization was better for reducing PMF and WC.

The case studies associated with Material optimization were further divided into those proposing Structural optimization (10 case studies) and Fluid dynamic optimization (5 case studies), where in Structural optimization, the mass of the product is reduced by optimizing the shape or the internal structure (e.g. Ahmed & Tsavdaridis, 2018), while in Fluid dynamic optimization the flow is rationalized, e.g. by increasing its turbulence (e.g. Agarski et al., 2017). Although limited to few overall case studies, this additional subdivision highlighted the major advantages of Fluid dynamic optimization compared to Structural optimization: Average impact (42% vs. 22%), GWP (44% vs. 21%), AP (32% vs. 16%), EP (33% vs. 12%), PMF (58% vs. 28%), RD (32% vs. 13%), WC (26% vs. 55%).

An alternative way to discuss Design for reducing waste strategy is instead in relation to the physical principle on which the solutions are based. To do this, the case studies were divided into those where the physical principle is modified (19 case studies) and the others (15 case studies). Some case studies associated with the first category are casting vs. additive manufacturing for the realization of a semi-finished product (Bekker & Verlinden, 2018), traditional vs. pulsator washing machine (Spreafico & Russo, 2020), roll paper vs. blower for drying hands (Joseph et al., 2015). From this classification are highlighted the advantages in percentage reduction of the impacts of the first category compared to the second one: Average impact (34% vs. 28%), GWP (37% vs. 26%), AP (35% vs. 20%), EP (32% vs. 24%), PMF (39% vs. 25%) and RD (45% vs. 7%).

This result is also useful for providing experimental and quantitative evidences to Kemp (2010) considerations about the link between eco-innovation and environmental sustainability. In this work, the author discussed the environmental advantages guaranteed by the application of different innovation strategies to eliminate the produced wastes. While Bossink (2013) hypothesized that the rethinking of the functioning of a device can reduce the wastes by limiting the provided evidences only for some specific application fields.

### Design for using renewable energies

The result associated with Design for using renewable energies confirmed the doubts raised by Horbach et al. (2015) about this strategy in implementing CE. Our study showed that the main strengths of this strategy are the reductions or RD and GWP. Both these reductions are primarily due to the use of solar and wind energy in large plants (e.g., Tannous et al., 2018; Yan et al., 2018), while, in smaller installations such as hydrogen-powered vehicles (e.g., Lajunen & Lipman, 2016) and biogas production by anaerobic digestion activated by sun (e.g., Wu et al., 2020) the impact reductions associated with this strategy were lower. In the analysed case studies, the reason provided about this influence of the scale factor are the higher impacts of the realization of technologies for exploiting renewable energies compared to fossil-fuelled plants. Finally, the obtained result is fully with the work of Elia et al. (2017) which associated a significant role in the use of renewable energies in the CE only in energy-intensive plants. However, that analysis excluded some environmental impact categories, including emissions to air, soil and water, material losses and resource depletion.

### Design for reuse

Only the detailed analysis of all the case studies associated with Design for reuse explained why this strategy obtained modest results. When Design for reuse is associated with building materials (e.g. Rios et al., 2015) or mechanical components (e.g. Postacchini et al., 2018), reductions in environmental impacts are on average between 36 and 74%. These values are therefore comparable or greater than Design for Recycling. However, the data extracted from some other case studies lowered the average reductions in all impact categories. The reason is the considerable amount of energy required to transport water to be reused, not present in freshwater systems. In these cases, the average environmental impact reduction is 17%. This conclusion provides quantitative evidences to the more qualitative observations provided by Voulvoulis (2018).

### Design for remanufacturing

**Fig. 4:**
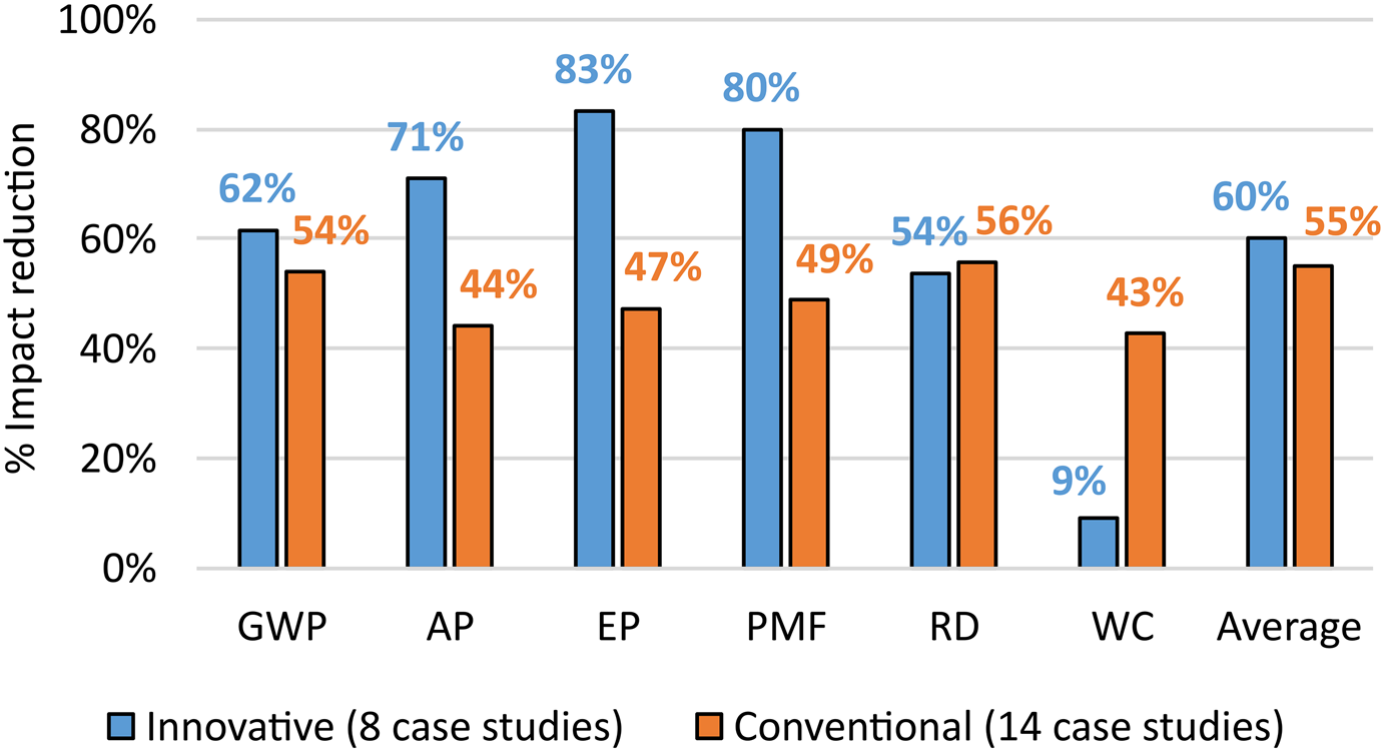
Percentage reductions of each environmental impact category associated with the two sub-strategies of Design for remanufacturing

The good results in reducing the environmental impacts in the case studies associated with Design for remanufacturing depend on the type of performed processing. This aspect clearly emerges by classifying the case study two sub-classes about conventional remanufacturing (14 case studies) and innovative remanufacturing (8 case studies), as shown in Fig. 4. Conventional remanufacturing mainly involves processes carried out with traditional machine tools for chip removal (e.g. Zhang & Chen, 2015). While innovative remanufacturing is based on laser processing (e.g. Leino et al., 2016) or on additive manufacturing (e.g. Wilson et al., 2014) to allow more focused repairs by reducing filler material and energy consumption.

Figure 4 clearly shows the advantages of innovative remanufacturing over conventional remanufacturing in reducing environmental impacts, especially of AP, EP and PMF. On the other hand, the impact reduction of WC (−34%) was against the trend and contributed to reduce the margin of advantage of the average impact (5%). At the application level, the two ways of proceeding clearly influence the Design for remanufacturing, by providing different solutions depending on the two cases, in all five phases of this discipline, i.e. design for reverse channel, design for environment, design for dismantling, design for lifecycle extension, design for improvement and design for appraisal (Charter & Gray, 2008). The results obtained with this classification provide quantitative evidence to the same theoretical comparison, previously discussed in Singhal et al. (2020).

### Design for recycling

**Fig. 5:**
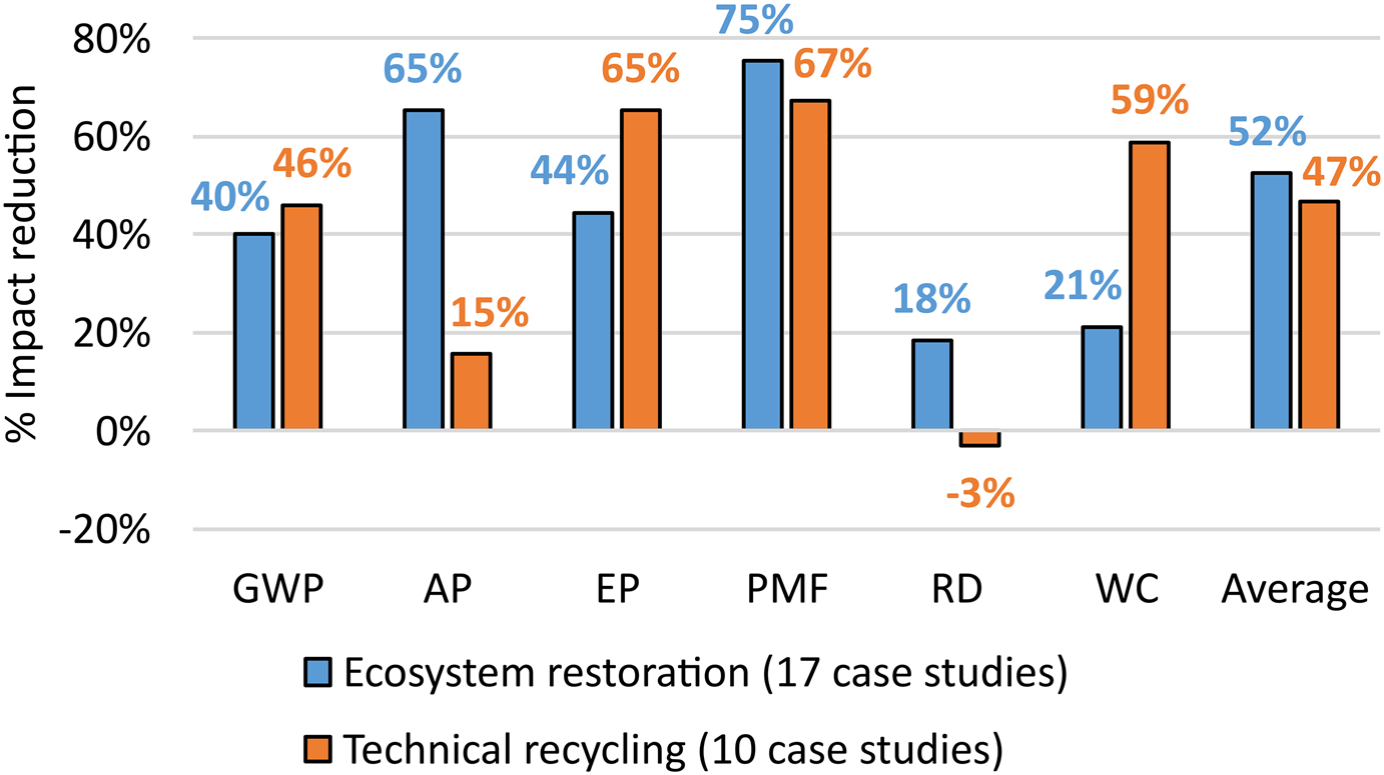
Percentage reductions of each environmental impact category associated with the two sub-strategies of Design for recycling

By classifying the case studies associated with Design for recycling in the two sub-strategies about ecosystem restoration (17 case studies) (Cherubini et al., 2015) and technical recycling (10 case studies) (Bertolini et al., 2016) (see Fig. 5), described in “Tested design strategies for circular economy”, the reasons of the results of this strategy can be comprehended.

Figure 5 shows that ecosystem restoration is, on average, better than technical recycling, which, however, allows a good average reduction of environmental impacts (i.e. 47%). The main advantages of the first sub-strategy, compared to the second, were found above all regarding AP (+ 50%), RD (+ 21%) and PMF (+ 8%). From the analysis of the case studies emerged the role of the recycling process. Both the sub-strategies are convenient for environmental sustainability, avoiding the introduction of virgin materials, but the technical recycling processes are generally more impactful than the others. This is because, in the second case, a more radical transformation of the waste, and also more energy-intensive, is required (e.g. de Souza Junior et al., 2020; Pintilie et al., 2016). However, this result is partial, because some authors in the literature (e.g. Convertino et al., 2013) highlighted other parameter to consider (e.g. local species richness, river basin extension, hydroperiod). Therefore, the provided assessment is starting point to be integrated with a more comprehensive analysis, even if still lacking in the literature.

### Design for energy recovery

**Fig. 6:**
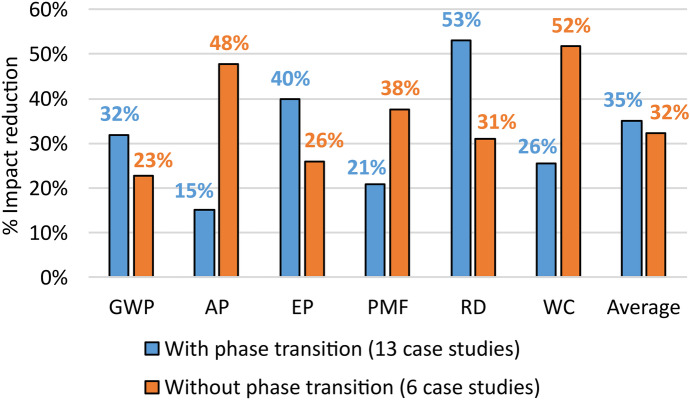
Percentage reductions of each environmental impact category associated with the two sub-strategies of Design for energy recovery

To discuss the results that have been associated with Design for energy recovery in this study, a further subdivision between systems that propose pure energy recovery (6 case studies) (e.g. Feng et al., 2014) from those that also introduce phase transition (13 case studies) (e.g. Cánovas et al., 2013) was provided (see Fig. 6).

Through the classification, both the sub-strategies were found to be beneficial for all the environmental impact categories (see Fig. 6). In addition, the phase transition in energy recovery was better for GWP, EP and RD. This result is useful for extending the advantages of this sub-strategy in environmental sustainability, previously provided by Spreafico (2021) also to implement CE. Liu et al. (2019), Boavida et al. (2020) and Vinodh et al. (2014) sustained the same conclusions but without supporting it with the assessment of the impacts trough standard categories, while Ozkeser (2018) limited the assessment to the GWP. However, in all these studies, the proposed evaluation is too aggregated, since different inventive principles are considered altogether and the considered case studies are few and too specific.

### Design for disposal

**Fig. 7:**
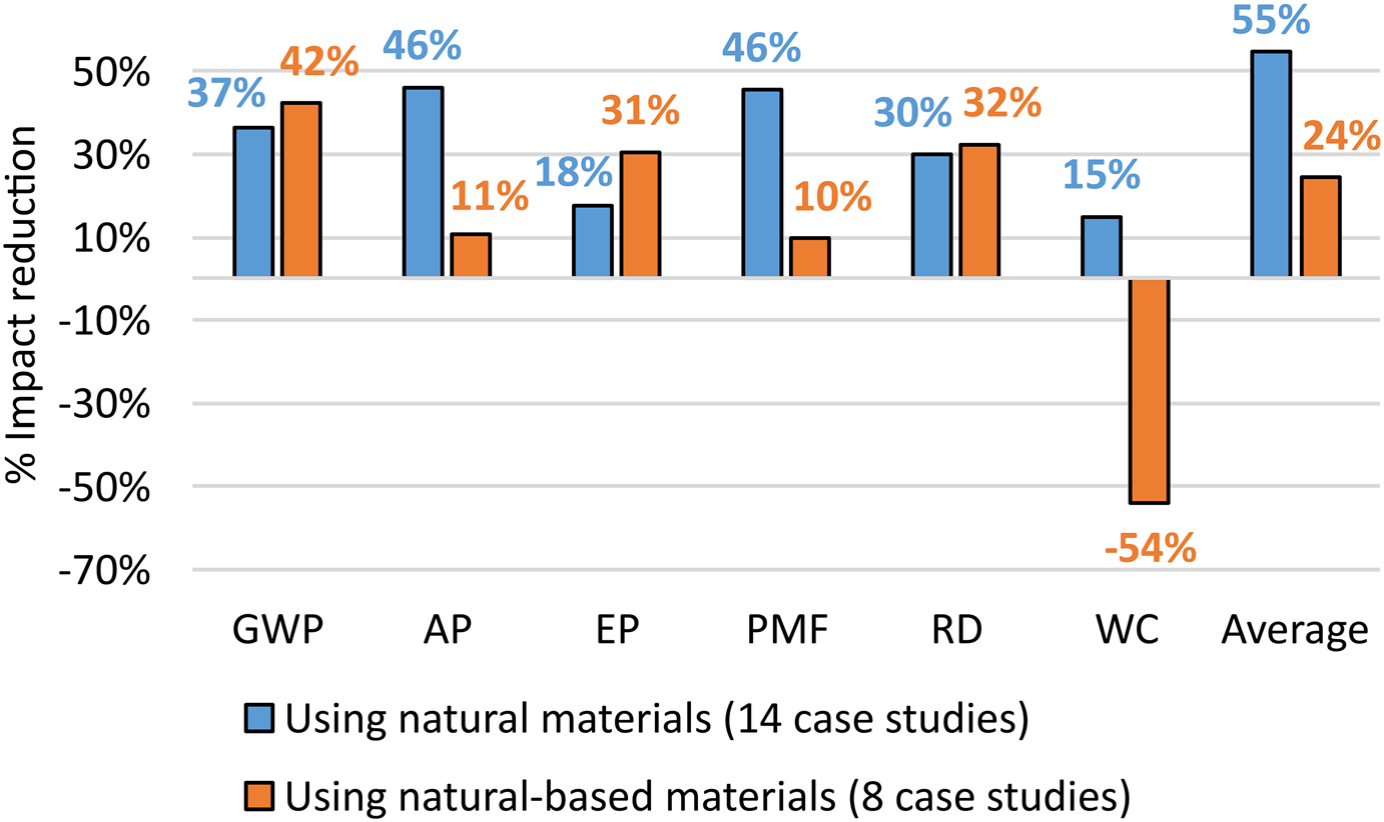
Percentage reductions of each environmental impact category associated with the two sub-strategies of Design for disposal

To adequately discuss the results obtained for the Design of disposal, the case studies referring to this strategy were further divided into its two sub-strategies, i.e. using natural materials (14 case studies) (e.g. Liu et al., 2019) and using natural-based materials (8 case studies) (e.g. Razza et al., 2015). The reductions in environmental impacts associated with these two sub-strategies were determined and reported in Fig. 7.

The distinction between the sub-strategies proved to be useful for better understanding the relationships between Design for disposal and the resulting reduction in environmental impacts (see Fig. 7). The greatest environmental advantages associated with this strategy were obtained in the case studies where natural materials have been introduced. They proved to be more sustainable than natural-based materials in reducing the average impact (+ 31%) and in almost all impact categories, except GWP (−5%), EP (−13%) and RD (−2%). In addition, the negative result obtained by natural-based material in WC (−54%) was also highlighted.

These results therefore confirm on a quantitative level some observations that have been made in the literature about the sustainability of the disposal of natural-based materials and to the tendency to prefer materials as natural as possible during this phase, although these observations are based on restricted application fields. For example, Hottle et al. (2013), with a review of LCA studies on bio-polymers, show that such materials are sustainable during production, while during disposal have some impacts similar to synthetic polymers. The conclusion is therefore to prefer biodegradable materials. This trend is recently being sought in food packaging, both for the realization of the packaging with natural materials such as chitosan, and with the increasing use of vegetable oils and acids as bactericides (Spreafico & Russo, 2021). Another field of application is in the construction of asphalts for road repair, where the use of unprocessed natural materials such as gravel and rocks in larger portions and the limitation of natural-based materials to targeted infill during maintenance are preferred (Landi et al., 2020).

### Design for recovering energy from waste

The results associated with this strategy confirmed the advantages of the implementation of CE and the research about the supporting design strategies. The analysis of the considered case studies also showed that the result does not change much when discriminating between different types of waste treatment. Pyrolysis (e.g., Vocciante et al., 2019) environmentally performed better than traditional incineration (e.g., Hossain & Poon, 2018), although the latter is significantly more widespread worldwide (Spreafico et al., 2021). The alternating results for the different impact categories of this strategy (see Fig. 2) show that there are some advantages for certain impact categories but their quantities nor comparable with the other CE options.

### Final considerations

In order to provide a further term of evaluation of the Design strategies for CE, Table 2 reports the standard deviations associated with the percentage reductions of the environmental impact categories for their derived sub-strategies, which average impact reductions as reported in Figs. 3, 4, 5, 6, and 7.

**Table 2:** Standard deviations associated with the percentage reductions of the environmental impact categories of the sub-strategies of the considered Design strategies for CE

**CE options**	**Design strategies**	**GWP**	**AP**	**EP**	**PMF**	**RD**	**WC**	**Average**
**Design for reducing wastes**	Dematerialization	31%	35%	29%	61%	25%	34%	33%
	Material optimization	30%	31%	37%	18%	20%	30%	28%
**Design for remanufacturing**	Innovative	24%	13%	9%	8%	11%	21%	24%
	Conventional	29%	26%	33%	38%	41%	25%	24%
**Design for recycling**	Ecosystem restoration	100%	68%	21%	138%	30%	73%	72%
	Technical recycling	90%	68%	72%	159%	105%	88%	81%
**Design for energy recovery**	With phase transition	27%	26%	32%	23%	46%	29%	28%
	Without phase transition	31%	40%	32%	13%	40%	20%	29%
**Design for disposal**	Natural materials	34%	46%	21%	33%	21%	37%	39%
	Natural-based materials	31%	17%	26%	39%	18%	28%	21%

**Fig. 8:**
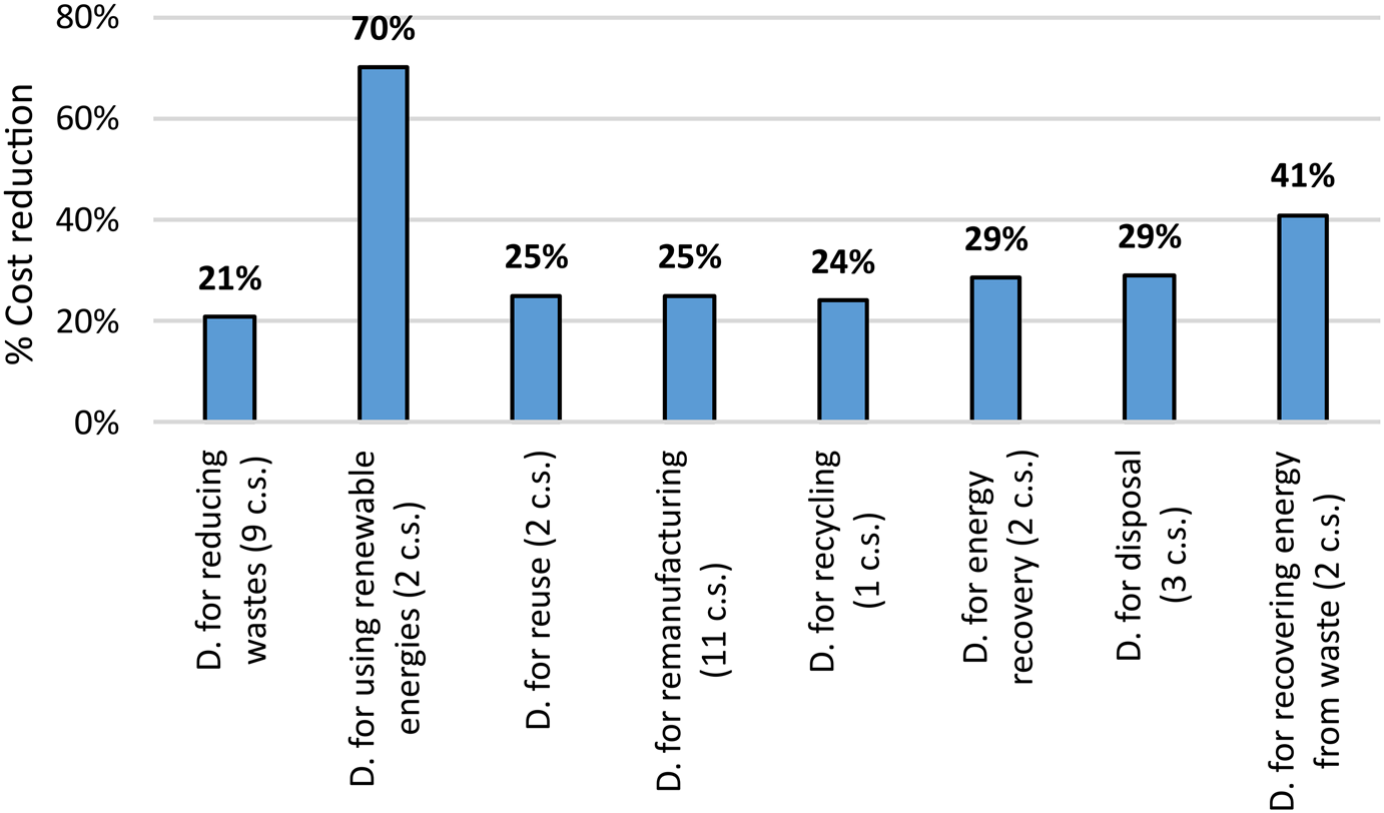
Percentage impact reductions of the average cost associated with each design (D.) strategy for CE resulted from the analysis of the considered case studies (c.s.)

On the other hand, Fig. 8 shows the result of the economic evaluation of the considered Design strategies for CE. For each of them, the average of the percentage reduction in the costs of the solutions obtained by their application compared to the baseline options is reported.

The result shown in Fig. 8 suffers from a major limitation compared to those relating to environmental impacts in Figs. 1, 2, 3, 4, 5, 6, and 7, i.e. the smaller number of considered case studies. This is because only a part of the considered articles also provides economic considerations. Consequently, the results associated with some strategies are based on a statistically insignificant number of sources (i.e. even fewer than three articles). In light of such limitations, this result should not therefore be used to compare the different strategies, ranking them on the basis of economic convenience. It is instead a confirmation also of the increased economic sustainability, as well as environmental, of the solutions associated with considered Design strategies for CE, compared to the other solutions.

Table 3 summarizes the observations that emerged from the discussion of the results obtained for all tested Design strategies for CE, shown in “Design for reducing wastes”, “Design for using renewable energies”, “Design for reuse”, “Design for remanufacturing”, “Design for recycling”, “Design for energy recovery”, “Design for disposal” and “Design for recovering energy from waste”. For each strategy, the advantages and the disadvantages for environmental sustainability were reported, i.e. how they were applied to achieve the greatest and least average reductions in environmental impacts, respectively, compared to the baseline scenario.

**Table 3:** Summary of the advantages and disadvantages of applying Design strategies for CE in the considered case studies

**Design strategies for CE**	**Advantages (good for)**	**Disadvantages (less good for)**
**Design for reducing wastes**	• Dematerialization • Changing the physical principle of operation of the system	• Material optimization, in particular structural optimization which was less sustainable than fluid dynamic optimization • Keeping the physical principle of operation of the system unchanged
**Design for using renewable energies**	Supplying large plants with renewable energies, in order to compensate for the environmental impacts deriving from the introduction of dedicated technologies	Supplying small plants with renewable energies
**Design for reuse**	Reusing mechanical products and components	Reusing natural resources (i.e. wastewater)
**Design for remanufacturing**	Using non-conventional techniques (e.g. laser)	Using conventional techniques (e.g. chip removal)
**Design for recycling**	Ecosystem restoration	Technical recycling
**Design for energy recovery**	Phase transition	Heat exchanger without phase transition
**Design for disposal**	Natural materials	Natural-based materials (e.g. bio-polymers)
**Waste to energy**	Pyrolysis	Incineration

### Perspectives and prospects

The results of this study can serve as a basis for some future research directions. The fact of having questioned the more common hierarchy of CE options, by introducing new categories of environmental impact, should be studied in greater depth. On the one hand, it could be useful to investigate the relationships between the CE options and the types of environmental impacts, by identifying which operations, necessary to implement a CE option, have the highest impacts, why and in what way. On the other hand, it is necessary to compare the hierarchy of the CE options with a hierarchy of importance of the categories of environmental impact, in order to confirm or reject the first one through a more reliable indicator, e.g. by using a weighted mean.

Furthermore, the heterogeneity of the evaluations of the CE options in relation to the characteristics of the product and the application field can be better formalized. In this case, the product requirements could be formalized in a more rigorous manner, for instance by using a systematic approach for their classification, such as Quality Function Deployment. Finally, a broader development that requires a deeper understanding of these issues could be the development of a design framework to suggest which design strategies could be applied to implement a CE option in a certain product with specific requirements, to guarantee, at the same time, the environmental sustainability.

## Conclusions

This study proposed the eco-assessment of some common design strategies to implement some CE options, by manually analysing 156 case studies of Comparative LCA from selected scientific articles. The results are the percentage reductions of different standard impact categories compared to baseline scenarios where CE is not implemented. The main limitations of this study concern the analysed case studies, which although in large numbers belong only to certain application areas. In addition, the number of considered case studies decreased notably for certain stratifications, e.g. by comparing the different design strategies referring to a CE option.

Through the obtained results, it was possible to hierarchize the design strategies for improving the environmental sustainability (RQ. 1), although the emerged hierarchy differs from that provided by some other studies. Considering the average of all impact categories, Design for remanufacturing produced the best options with impact reductions by 53%, followed by Design for recycling (45%), while Design for recovering energy from waste was the worst option, increasing the impacts by 83%. Considering instead the different impact categories, a certain variability emerged, as well as between the results of the different case studies and those within the Design strategies (with standard deviations between 21 and 81%). Finally, the economic sustainability of the solutions arising from the Design strategies for CE is also considered, although considering a smaller number of case studies.

Nevertheless, by deeply analysing the considered case studies, some motivations for the advantages and disadvantages for environmental sustainability of the different Design strategies for CE were also identified. They depend by the specific characteristics of the products and the application fields, thus confirming RQ. 2.

## Data Availability

The datasets generated during and/or analysed during the current study are available from the corresponding author on reasonable request.

## Appendix

**Table 4:** Information about the considered articles, the case studies, the associated strategies and sub-strategies and the percentage reductions of the environmental impacts and costs (where 1 = design for reducing waste, 2 = design for using renewable energies, 3 = design for reuse, 4 = design for remanufacturing, 5 = design for recycling, 6 = design for energy recovery, 7 = design for disposal, 8 = design for recovering energy from waste, 1A = dematerialization, 1B = material optimization, 4A = innovative, 4B = conventional, 5A = ecosystem restoration, 5B = technical recycling, 6A = with phase transition, 6B = without phase transition, 7A = natural materials, 7B = natural-based materials)

**Articles**	**Case studies**	**System 1**	**System 2**	**Design strategies**	**Sub-strategies**	**Percentage reductions**							
						**Impact reduction**							**Cost reduction**
						**GWP**	**AP**	**PMF**	**EP**	**WC**	**RD**	**Average impact**	
**Adghim et al. **(2020)	Dairy waste processing	Conventional (output: fertilizer)	Anaerobic digestion (output: fertilizer + electricity)	5	5A	−25%	−49%		−18%	0%	−30%	−24%	
**Agarski et al. **(2017)	Catalyst synthesis process	Catalyst fluid	Ultrasonic aerosol	1	1B	−86%	−80%	−83%	−50%		−70%	−74%	–18%
**Ahmed and Tsavdaridis **(2018)	Buildings	Composite precast slab	Mass optimization	1	1B	−65%						−65%	–13%
**Ahmed and Tsavdaridis **(2018)	Buildings	Cofradal260 slab	Mass optimization	1	1B	−60%						−60%	–46%
**Akhshik et al. **(2017)	Engine beauty cover	Fiberglass	Bio-based	7	7B	−57%	−32%	−62%	−64%	54%		−32%	
**Alvarez et al. **(2020)	Textile process	Conventional	With wastewater reuse	3		23%	13%	−3%	−27%			1%	
**Asdrubali et al. **(2015)	Concentrated solar power cooling	Liquid	Air	1	1A	10%	10%	10%	10%	10%	10%	10%	
**Bailis et al. **(2013)	Pyrolysis	None	With cogeneration	8		−325%	−10%		−10%			−115%	
**Bartolozzi et al. **(2017)	Distric energy heating	Conventional	Geothermal	2		−17%	38%	60%	29%	109%	−46%	29%	
**Batuecas et al. **(2019)	Olive oil production	Disposal on soil	Anaerobic digestion + syngas production + energy recovery	5	5A	−45%	−44%			−42%	−61%	−48%	
**Bekker and Verlinden **(2018)	Steel printing	Casting	Additive manufacturing	1	1B	2%	1%	3%				2%	
**Bertolini et al. **(2016)	Milk packaging	PET	Multilayer cartoons and plastic	7	7B	−37%	−24%		−6%			−22%	
**Bezama et al. **(2013)	Municipal waste treatment	Landfill	Landfill with energy recovery	5	5A	−39%						−39%	
**Bezama et al. **(2013)	Municipal waste treatment	Landfill	Anaerobic digestion with energy recovery (method 1)	5	5A	−100%						−100%	
**Bezama et al. **(2013)	Municipal waste treatment	Landfill	Anaerobic digestion with energy recovery (method 2)	5	5A	−100%						−100%	–34%
**Biswas and Rosano **(2011)	Air conditioning compressor	None	Remanufacturing	4	4B	−89%				−90%	−90%	−90%	
**Boland and Unnasch **(2014)	Car grill shutter	70% Polypropylene + 30% glass fiber	70% Polypropylene + 30% cellulose fiber	7	7B	−21%					9%	−6%	–34%
**Bonamente and Aquino **(2019)	Ground heat pump	Traditional	Phase change + condensing	6	6A	−17%	−15%					−16%	–15%
**Boustani et al. **(2010)	Dishwasher	New model	Remanufactured model	4	4B						−60%	−60%	
**Cánovas et al. **(2013)	Heat pump residential heating	Heat pump single state	Heat pump double stage (air/water composition 1)	6	6A	−59%						−59%	
**Cánovas et al. **(2013)	Heat pump residential heating	Heat pump single state	Heat pump double stage (brine/water composition 1)	6	6A	−71%						−71%	
**Cánovas et al. **(2013)	Heat pump residential heating	Heat pump single state	Heat pump double stage (air/water composition 2)	6	6A	−56%						−56%	
**Cánovas et al. **(2013)	Heat pump residential heating	Heat pump 1 state	Heat pump (brine/water composition 2)	6	6A	−71%						−71%	
**Carvalho et al. **(2018)	Fryer	Stovetop deep frying	Hot-air frying (model 1)	7	7A	−92%						−92%	
**Carvalho et al. **(2018)	Fryer	Stovetop deep frying	Hot-air frying (model 2)	1	1A	−92%						−92%	
**Castell et al. **(2013)	Brick insulation	Brick	Brick with phase change	6	6A	−13%						−13%	
**Chen et al. **(2016)	PET production	New PET	Biobased recycled PET	5	5A	−21%					−22%	−22%	
**Cherubini et al. **(2015)	Manure elimination	Conventional	Composting	5	5A	1%	8%		0%		5%	4%	–50%
**Choe et al. **(2013)	Ion exchange water treatment	Non-selective (using granular media filter)	Perchlorate selective (using gaseous catalyst filter)	1	1A	−30%	−70%		−93%		−30%	−56%	
**Colazo et al. **(2015)	Solid waste anaerobic digestion	Wet	Dry	7	7A	−18%	−20%	−17%	−17%	−30%		−20%	
**de Souza Junior et al. **(2020)	Polystyrene production	Virgin	Recycled	5	5B	−39%	−13%	3%	62%	23%	−24%	2%	
**Dias et al. **(2013)	Diesel engine	New model	Remanufactured model	4	4B	−74%						−74%	
**Dong et al. **(2018)	Waste elimination	Ladfill	Incinerator with energy recovery	8		−81%	−250%					−166%	–65%
**Dong et al. **(2018)	Waste elimination	Ladfill	Landfill with energy recovery	5	5A	−73%	−200%					−137%	–24%
**dos Santos Pegoretti et al. **(2014)	Car acoustic components	Poliurethane	Recycled cotton	5	5A	−1%	−50%	−44%				−32%	
**Eranki et al. **(2017)	Irrigation	Flood	Drip	1	1B	−20%	−17%	−32%	−16%		−13%	−20%	–23%
**Feng et al. **(2014)	Flue gas desulphurization	Coal firing	Combined cycle	6	6B	−3%	−70%	−38%	−35%	−80%	−45%	−45%	
**Forchino et al. **(2017)	Aquaponics	Raft (with polystyrene)	With media filled (gravel substrate)	7	7A	15%	18%		4%	0%	13%	10%	
**García-Alcaraz et al. **(2020)	Wood wine barrel disinfection	Water vapour + SO2	Ozone	7	7B	−47%	−48%					−47%	
**Goulet et al. **(2017)	Inhaler filling	Mechanic/pneumatic	Electric nebulizer	1	1B	−50%						−50%	
**Guerra et al. **(2014)	Sugarcane production	Vapour cycle	Regenerative vapour cycle	6	6B	−6%	−6%	−6%		−6%		−6%	
**Hengen et al. **(2014)	Acid mine drainage treatment	Active	Passive (using biogechemical)	7	7A	−78%		−42%			−44%	−55%	
**Hengen et al. **(2014)	Acid mine drainage treatment	Active (syntetic)	Passive (using natuarl biogechemical)	7	7A	−78%		−42%			−44%	−55%	
**Hossain and Poon **(2018)	Disposing wood waste	Landfill	Relizing polymer-based material	5	5A	−27%					171%	72%	
**Hossain and Poon **(2018)	Disposing wood waste	Landfill	Incineration	8		1020%					805%	913%	
**Hossain and Poon **(2018)	Disposing wood waste	Landfill	Realizing cement-based material	5	5A	−70%					−2%	−36%	
**Jachimowski and Nitkiewicz **(2019)	Water disinfection	Gaseous chlorine	UV	2		−95%	−93%				−94%	−94%	
**Jones et al. **(2018)	Water disinfection	Gaseous chlorine	UV	2		18%	29%	27%	36%	15%		25%	
**Joseph et al. **(2015)	Hand dryer	Roll paper	Blower	1	1A	−58%					−21%	−39%	–22%
**Kalbusch and Ghisi **(2016)	Tap	Conventional	Water saving	1	1B	−14%	0%			−26%	−13%	−14%	
**Karami et al. **(2015)	Buidlings insulation	Standard panales	Vacuum panels	1	1A	−6%					26%	10%	–26%
**Katsiropoulos et al. **(2019)	Helicopter’s Canopy production	Cold diaphragm forming	Autoclave	1	1A	7%						7%	–19%
**Katsiropoulos et al. **(2019)	Helicopter’s Canopy production	Resin transfer molding	Autoclave	1	1A	11%						11%	–16%
**Khalil **(2018)	Carbon fiber recycling	Thermolysis	Solvolysis (with supercritical water)	7	7B	2%	4%	4%	1%			3%	
**Klemeš **(2012)	Wastewater	Landfill disposal	Reuse wastewater	3						−30%		−30%	–5%
**Krystofik et al. **(2014)	Wastre printer cartridge	Landfill disposal	Remanufacturing	4	4B	−10%	−11%	−11%	−14%	−18%	−9%	−12%	–15%
**Krystofik et al. **(2014)	Waste printer cartridge	Landfill disposal	Refilling	3		−20%	−24%	−24%	−32%	−37%	−20%	−26%	
**Kwofie and Ngadi **(2017)	Rice parboiled system	Traditional	With heat recirculation	6	6B	−83%	−92%	−69%				−81%	–60%
**Lajunen and Lipman **(2016)	Autobus	Diesel	Fuel cell hydrogen	2		9%						9%	
**Landi et al. **(2020)	Asphalt production	Traditional	Waste tire fiber reinforced	4	4B	−25%	−31%	−31%				−29%	
**Landi et al. **(2020)	Asphalt production	Traditional	Cellulose reinforced	7	7B	−10%	−10%	−11%				−10%	
**Leal et al. **(2020)	Street slope repair	Rock fill	Fibre reinforced soil	7	7A	−75%						−75%	
**Leal et al. **(2020)	Street slope repair	Rock fill cementification	Electro-osmosys	4	4B	−90%						−90%	
**Leino et al. **(2016)	Waste engine	Landfill disposal	Remanufacturing	4	4A	−45%					−36%	−41%	
**Li et al. **(2018)	Magnesium production	Single phase	Two phases	6	6A	5%	20%					12%	
**Ling-Chin et al. **(2016)	Marine power	Conventional	Retrofitted	2		−3%	−3%	−3%				−3%	
**Liu et al. **(2013)	Dam	Concrete	Concrete filled with rocks	7	7A	−63%		−50%			−53%	−55%	–50%
**Liu et al. **(2014)	Diesel engine	Landfill disposal	Remanufacturing	4	4B	−75%	−25%				−66%	−55%	
**Liu et al. **(2016)	Cylinder head	Landfill disposal	Remanufacturing	4	4A	−67%	−62%		−85%			−71%	
**Liu et al. **(2019)	Toilet flushing	Traditional	With seawater	7	7A	−10%	−16%	−21%				−16%	
**Lombardi et al. **(2017)	Sewage sludge elimination	Wet oxidation	Compostable with composition 1	5	5A	176%	−6%		14%		−41%	36%	
**Lombardi et al. **(2017)	Sewage sludge elimination	Wet oxidation	Compostable with composition 2	5	5A	170%	−4%		14%		−48%	30%	
**Lu et al. **(2017)	Electronic waste	Landfill disposal	Recycling	5	5B	−17%	−33%					−25%	
**Mohammed et al. **(2016)	NOx elimination	Landfill disposal	Recycling to obtain fertilizer	5	5A	−93%	−181%		−32%			−102%	
**Mondello et al. **(2017)	Waste elimination	Composting with UREA	Composting with insects	7	7A	−28%	−56%		−55%		−50%	−47%	
**Mondello et al. **(2017)	Wood waste	Landfill disposal	Incineration	8		−57%	−149%		−118%		−49%	−93%	
**Mondello et al. **(2017)	Wood waste	Landfill disposal	Obtaining a compost	5	5A	−92%	−75%		−87%		−78%	−83%	
**Mondello et al. **(2017)	Wood waste	Landfill disposal	Obtaining biogas	5	5A	−95%	−0.85427136		−93%		−86%	−90%	
**Montazeri and Eckelman **(2018)	Wood coating	Conventional (thermal)	UV-curing bio-based	2		−40%					−51%	−46%	–17%
**Morris **(2017)	Wood waste biomass	Landfill disposal	Incinerator with energy recovery	8		600%	−29%		−75%			165%	
**Morris **(2017)	Wood waste biomass	Landfill disposal	Recycling to obtain paper pulp	5	5A	−250%	−29%		−400%			−226%	
**Morris **(2017)	Wood waste	Landfill disposal	Obtaining reconstituted wood	5	5B	−275%	−43%		−450%			−256%	
**Mu et al. **(2010)	Lignocellulosic Ethanol Production	Biological	Thermal with energy recovery	6	6B	−29%				−69%	−17%	−38%	
**Nabavi-Pelesaraei et al. **(2017)	Urban waste	Landfill disposal	Recycling	5	5B	−50%	−60%		−40%			−50%	
**Ni et al. **(2020)	Thermal energy storage	Conventional (within tank)	Aquifer (option 1)	2		−56%	−26%	−60%				−47%	
**Ni et al. **(2020)	Thermal energy storage	Conventional (within tank)	Aquifer (option 2)	2		−50%	−16%	−50%				−40%	
**Oh et al. **(2019)	Lightweight ship realization	Conventional (glass fibre and resin)	Lightweight design by reducing resin	1	1B	−26%						−26%	
**Opher and Friedler **(2016)	Wastewater	Sewer disposal	Obtaining drinking water	5	5B	−24%	−24%	−23%	−23%	−24%	−23%	−24%	
**Opher and Friedler **(2016)	Wastewater	Sewer disposal	Reusing water for irrigation	3		−24%	−24%	−23%	−24%	−24%	−23%	−24%	
**Opher and Friedler **(2016)	Wastewater	Sewer disposal	Reusing water for industrial purposes	3		−23%	−23%	−28%	−23%	−23%	−29%	−25%	
**Oró et al. **(2012)	Energy storage for solar power plants	With molten salts	With phase change materials	6	6A	−58%	−58%	−58%	−58%	−58%	−58%	−58%	
**Ozbilen et al. **(2013)	Hydrogen production	Stream reforming	Solar-based electrolysis	2		−80%	−46%					−63%	–15%
**Ozoemena et al. **(2018)	Wind turbine transmission	Direct driving gearbox	Driven permanent magnet	1	1A	−13%	−36%		−47%			−32%	
**Ozoemena et al. **(2018)	Wind turbine	Conventional	Carbon fibre optimization	1	1B	6%	2%		0%			3%	
**Paccanelli et al. **(2015)	Nitrogen recovering for manure digestion	No treatment	Biological anaerobic	7	7A	5%	−2,80%	1%	−62%			−15%	
**Paccanelli et al. **(2015)	Nitrogen recovering for manure digestion	No treatment	With bacteria	7	7A	−29%	−2,90%	−1%	−60%			−23%	
**Pardo and Zufía **(2012)	Food conservation	Thermal pasteurization with water/steam injection	Thermal pasteurization with microwave	1	1B	−36%	17%		10%	−24%	−36%	−14%	
**Pardo and Zufía **(2012)	Food conservation	Thermal pasteurization with water/steam injection	Autoclave non-thermal pressurization with gas	1	1A	−29%	11%		78%	−24%	−29%	1%	
**Peng et al. **(2017)	Impeller	Disposal	Remanufacturing with additive manufacturing	4	4A	−65%			−75%		−64%	−68%	
**Peters **(2016)	Waste folder inserter machine	Landfill disposal	Remanufacturing	4	4B	−23%						−23%	–12%
**Pintilie et al. **(2016)	Wastewater	Sewer disposal	Obtaining drinking water	5	5B	70%	159%		−5%	−176%	152%	40%	
**Poinssot et al. **(2014)	Nuclear plant	Open fuel cycle	Closed fuel cycle	5	5B	−3%	−15%		−2%			−7%	
**Postacchini et al. **(2018)	Waste honey jars	Disposal	Reusing	3		−74%	−80%					−77%	–35%
**Pourzahedi and Eckelman **(2015)	Silver nanoparticles production	Spray pyrolysis	Reactive magnetron	1	1A	−77%	−78%		−76%		−78%	−77%	
**Pourzahedi and Eckelman **(2015)	Silver nanoparticles production	Spray pyrolysis	Arc plasma	1	1A	−74%	−75%		−76%		−76%	−75%	
**Pranjić et al. **(2018)	Contaminated soil treatment	Landfill disposal with biological effects	Incineration	8		88%	88%	87%	88%	84%	84%	87%	
**Razza et al. **(2015)	Foamed packaging	Synthetic	Bio-based and biodegradable	7	7B	−60%	8%		30%		−50%	−18%	
**Rew et al. **(2018)	Snow removal airlift	Mechanical	Conductive asphalt (con graphite and electric current)	1	1A	−28%						−28%	
**Rios et al. **(2015)	Building material (steel frame)	Wood (monouse)	Reused steel	3		−36%				−89%	−40%	−55%	
**Scalbi and Masoni **(2015)	Transistor cooling	Nanofluid single stage	Nanofluid double stage	6	6A	−24%	−3%	−3%	−13%			−11%	
**Schakel et al. **(2014)	Co-firing plants with carbon capture and storage	Supercritical pulverized coal	Integrated gassification combined cycle	6	6B	−6%						−6%	–12%
**Schau et al. **(2012)	Alternator	Standard	Lighweight	1	1B	−4%	−13%		−19%		−2%	−10%	–12%
**Schau et al. **(2012)	Alternator	Standard	Ultra-lightweight	1	1B	−3%	−10%		−16%		−1%	−8%	
**Schreiber et al. **(2019)	Wind turbine transmission	Direct driving gearbox	With permanent magnent	1	1A	−42%		−45%		−50%		−46%	
**Schulte et al. **(2021)	Medical catheteres	Disposal	Remanufacturing	4	4B	−50%						−50%	
**Shen et al. **(2010)	PET bottles	Novel	With recycled PET	5	5B	−25%	−40%	−160%	−8%			−58%	–40%
**Shi et al. **(2015)	Waste diesel engine	Disposal	Remanufacturing (option 1)	4	4B	−15%	−50%				−61%	−42%	
**Shi et al. **(2019)	Waste diesel engine	Disposal	Remanufacturing (option 2)	4	4A	−19%						−19%	
**Simion et al. **(2013)	Building	With natural inert	With rubble	5	5B	−84%	−70%	−81%	−70%		−80%	−77%	
**Spreafico and Russo **(2020)	Washing machine	Conventional	Pulsator	1	1B	−51%						−51%	
**Sproesser et al. **(2015)	Welding	Gas	Laser	1	1A	−50%	−47%					−48%	
**Stasiulaitiene et al. **(2016)	Gaseous pollutants filtration	Wet flue gas catalysts	Plasma	1	1A	−53%	−68%		−80%			−67%	
**Sun et al. **(2019)	Car body structure	Steel	Advanced High Strength Steel	1	1B	5%					10%	8%	
**Tagliaferri et al. **(2019)	Additive manufacturing	Fused deposition method	Selective laser syntering	1	1A	−33%	−47%				−37%	−39%	
**Tannous et al. **(2018)	Street lighting	Grid connected	Solar powered	2		−70%	−72%		−75%		−84%	−75%	
**Torres-Carrillo et al. **(2020)	Foundry	Casting	Selective laser melting	1	1A	−6%	−4%					−5%	
**Vignali **(2017)	Domesting heating	Conventional boiler	Condensing boiler	6	6A	−17%	−29%		−48%		−10%	−26%	–25%
**Villanueva-Rey et al. **(2018)	Viticulture	Conventional	Biologic (with compostable fertilizer)	7	7A	−61%	−60%		−84%			−68%	
**Villanueva-Rey et al. **(2018)	Wiring	Standard	Lightweight	1	1B	−56%	−60%	−60%		−55%	−57%	−58%	
**Vocciante et al. **(2019)	Phyto disposal	Disposal on soil	Incineration	8		−20%						−20%	
**Vocciante et al. **(2019)	Phyto disposal	Disposal on soil	Fast pyrolysis	8		−40%						−40%	
**Walker et al. **(2015)	Car	Gasoline	Fuel cell hydrogen	2		−67%					−52%	−59%	
**Wang et al. **(2019)	Biomass gassification	Standard	Reusing waste water	3		−7%						−7%	
**Warsen et al. **(2011)	Waste vehicle transmission	Disposal	Remanufacturing	4	4B	−31%	−36%		−40%		−33%	−35%	
**Wilson et al. **(2011)	Waste engine parts	Disposal	Remanufacturing	4	4A	−92%						−92%	–18%
**Wilson et al. **(2014)	Waste turbine blades	Disposal	Remanufacturing with laser deposition	4	4A	−45%					−36%	−41%	
**Wu et al. **(2020)	Biogas production from manure digestion	Gas pumping	Algae activated by sun (option 1)	2		−31%						−31%	
**Wu et al. **(2020)	Biogas production from manure digestion	Gas pumping	Algae activated by sun (option 2)	2		−25%						−31%	
**Xia et al. **(2020)	Waste concrete structures	Disposal	Recycling	5	5B	−13%					−10%	−12%	
**Xia et al. **(2020)	Waste concrete structures	Disposal	Reusing	3		−5%					−6%	−5%	–31%
**Xiao et al. **(2018)	Waste loading machines	Disposal	Remanufacturing	4	4B	−72%	−79%	−78%			−52%	−70%	–80%
**Yan et al. **(2018)	Potable water production	Centralized	Decentralized + using wind energy	2					−47%	−53%	−96%	−65%	
**Young et al. **(2019)	CO_2_ sequestration	None	Postcombustion	6	6A	−7%	−6%	−1%		7%		−2%	
**Zakuciová et al. **(2020)	CO_2_ sequestration	Activated carbon sieve by adsorption	Gas oxidizers	1	1A	−73%	−36%	−36%				−48%	
**Zanchi et al. **(2016)	Car door	Aluminum	Ligheight aluminum	1	1B	−7%						−7%	–18%
**Zea Escamilla et al. **(2018)	Buildings	Bricks	Bamboo	7	7A		−107%					−107%	–13%
**Zea Escamilla et al. **(2018)	Buildings	Bricks + concrete	Bamboo	7	7A		−106%					−106%	–46%
**Zea Escamilla et al. **(2018)	Buildings	Concrete hollow	Bamboo	7	7A		−108%					−108%	
**Zhang and Chen **(2015)	Waste diesel engine	Disposal	Remanufacturing	4	4B	−69%						−69%	
**Zhang et al. **(2010)	Wastewater	Disposal	Reusing in industrial process	3						−13%		−13%	
**Zhang and Xu **(2020)	Power plants cooling	Heat exchanger	Heat exchanger with encapsulated phase-change materials (option 1)	6	6A	−13%					−72%	−43%	
**Zhang and Xu **(2020)	Power plants cooling	Heat exchanger	Heat exchanger with encapsulated phase-change materials (option 2)	6	6A	−10%					−72%	−41%	
**Zhang et al. **(2020)	Waste diesel engine	Disposal	Remanufacturing using laser cladding	4	4A	−80%						−80%	
**Zheng et al. **(2019)	Waste diesel engine	Disposal	Remanufacturing using arc spraying	4	4B	−78%	−77%	−75%	−88%	−20%	−75%	−69%	
**Zheng et al. **(2019)	Diesel engine	New model	Remanufacturing using laser cladding	4	4A	−80%	−80%	−80%	−90%	−9%	−79%	−70%	
**Zhu et al. **(2015)	Ethanol synthesis	Direct thermochemical conversion	Indirect thermochemical conversion	6	6B	−10%	−23%		−17%			−17%	
